# Comparison of the effects of pea protein and whey protein on the metabolic profile of soccer athletes: a randomized, double-blind, crossover trial

**DOI:** 10.3389/fnut.2023.1210215

**Published:** 2023-09-22

**Authors:** Luiz Lannes Loureiro, Tathiany Jéssica Ferreira, Fábio Luiz Candido Cahuê, Victor Zaban Bittencourt, Ana Paula Valente, Anna Paola Trindade Rocha Pierucci

**Affiliations:** ^1^DAFEE Laboratory, Institute of Nutrition, Federal University of Rio de Janeiro, Rio de Janeiro, Brazil; ^2^CNRMN, Structural Biology, Institute of Medical Biochemistry, Federal University of Rio de Janeiro, Rio de Janeiro, Brazil

**Keywords:** NMR spectroscopy, plant protein, dietary supplements, amino acids, recovery

## Abstract

**Introduction:**

Pea protein (PP) concentrate is a plant-based alternative to animal protein sources, such as whey protein (WP). In addition to its valuable amino acid composition, PP has a low environmental impact, making it a sustainable, nutritious, and viable alternative for enhanced sports performance, such as in soccer. PP Therefore, this study aimed to evaluate the effects of PP and WP supplementation on biochemical and metabolic parameters in soccer players.

**Methods:**

Twelve male under-20 soccer players were included in this double-blind, randomized crossover intervention study. For 10 consecutive days, each participant received either 0.5 g/kg of the PP or WP supplementation after training, starting 7 days before the test game, and continuing until 2 days after. After a 4-day washout period, the athletes switched groups and the intervention was restarted. Blood samples were collected before and after the game, as well as 24 h, 48 h, and 72 h intervals thereafter. Creatine kinase (CK), aspartate transaminase, alanine transaminase (ALT), lactate (LA), urea, creatinine, and uric acid were analyzed using commercial kits. Exploratory metabolic profiling of the serum samples was performed using nuclear magnetic resonance spectroscopy.

**Results:**

A comparison of biochemical markers showed that the PP group had lower CK in the post-game moment, 24 h, and 48 h. Lower LA in the post-game moment, and lower ALT in the post-game moment and at 24 h. Of the 48 metabolites analyzed, 22 showed significant differences between the time points, such as amino acids, ketone bodies, and glucose metabolism. Glutamate and lactate levels significantly increased between the pre- and post-game moments in the WP group. After the game, the WP group exhibited reduced levels of metabolites such as arginine and taurine, whereas no such change was observed in the PP group. There was no difference in metabolites 72 h after the game.

**Conclusions:**

Despite the slight advantage of the PP group in specific biochemical markers, these differences are not sufficient to justify the choice of a particular type of protein. However, the results highlight the viability of plant protein as a potential alternative to animal protein without compromising athletic performance or recovery.

## Introduction

Soccer is characterized by intermittent high-intensity physical exercise, involving a combination of kicks, jumps, and sprints (1). Intense and prolonged exercise promotes an increase in oxidative stress. Several studies have shown an association between oxidative stress, cell damage, inflammation, and decreased physical performance, particularly evident after consecutive matches (2–5).

Soccer athletes can participate in two to three weekly matches in national and international competitions. However, Viana-Gomes et al. (3) showed that the recovery interval between soccer matches is insufficient to help athletes recover and reduce oxidative stress. Thus, it is possible to postulate that subsequent matches can contribute to increased oxidative stress and cellular damage maintenance, which can also induce muscle injury.

Several studies have evaluated the effect of nutritional supplementation on the reduction of muscle damage (6–8), oxidative stress (9, 10), and improved performance (8, 11, 12). Among the existing nutrients and bioactive compounds, protein is highlighted in science, with studies evaluating its effectiveness (13–15).

Proteins play several roles in muscle recovery, including increasing muscle protein kinetics and mitochondrial biogenesis, activating signaling proteins in the protein synthesis cascade, reducing unwanted inflammatory responses, and contributing to strength recovery (16, 17). Poulios et al. (18) conducted a systematic review by selecting ten studies including seven studies about soccer. They verified the positive effects of protein supplementation on the reduction of inflammatory markers, oxidative stress, muscle damage, and performance improvement.

The main proteins studied in the context of human performance are from animal sources [e.g., whey protein (WP)] (19). WP is a common choice for protein supplementation among athletes due to its rich content of branched-chain amino acids (BCAAs), exceptionally high leucine content, rapid digestibility, and its ability to stimulate the synthesis of muscle protein (20). Additionally, plant proteins have gained prominence in sports science, exhibiting positive effects on performance and recovery (20, 21). Animal and plant-based proteins are also commonly described by their ability to influence postprandial amino acid profiles and their capacity to regulate rates of muscle protein synthesis post-ingestion (22).

Pea protein (PP) is a plant-based protein considered high-quality, that includes four major classes (globulin, albumin, prolamin, and glutelin), in which globulin and albumin are major storage proteins in pea seeds (23). In addition, it is composed of essential amino acids, limited only to sulfur amino acids such as methionine and cysteine (24, 25) and a source of bioactive small peptides (26). A study conducted by Banaszek et al. (20) revealed that both WP and PP could promote similar strength, performance, body composition, and muscular adaptations following an eight-week High-Intensity Functional Training program. The similarity of the results observed between the two types of proteins can be attributed to the amino acid profile and to the digestibility of the proteins (20). However, few studies have demonstrated the effect of PP on reducing muscle damage in competitive team sports such as soccer.

While targeted analysis of biochemical markers related to the immune and antioxidant systems and cell and tissue damage may provide a promising approach for evaluating exercise and nutritional interventions, assessment of the non-targeted metabolite profile may provide a more comprehensive understanding of the overall metabolic state of individual athletes (27). Metabolomics is a powerful tool that helps elucidate complex mechanisms and exercise-related disorders (28, 29), such as to evaluate the acute effects of hydration, nutritional strategies to manage oxidative stress, and inflammation and immune response, prevent and manage injuries and to determine how training can impact the metabolome over long or short periods (30).

Some studies have observed metabolic changes resulting from participation in soccer matches (31–33). Quintas et al. (31) observed metabolomic profiling allowed the detection of subtle changes in the urinary metabolome, which were associated with the external training load in professional football players throughout one season. Partial least squares (PLS) models identified steroid hormone metabolites, hypoxanthine metabolites, acetylated amino acids, intermediates in phenylalanine metabolism, tyrosine, tryptophan metabolites, and riboflavin among the most relevant variables associated with external load. Pitti et al. (32) used nuclear magnetic resonance (NMR) to assess the metabolomic profile after a soccer match. They identified an increase of metabolites related to fatigue, cell damage, and inflammation, such as amino acids ornithine, tyrosine, phenylalanine, and histidine, putrescine, succinate, and creatinine.

Therefore, the objective of this study was to conduct a comparative evaluation to verify whether 10-day supplementation with PP and WP would have a distinct influence on biochemical and metabolomic alterations up to 48 h after a simulated soccer match (game) in professional athletes under-20 (U-20). The hypothesis of the present study is that both WP and PP can reduce muscle damage in competitive team soccer sports.

## Materials and methods

### Study design

**Figure 1:**
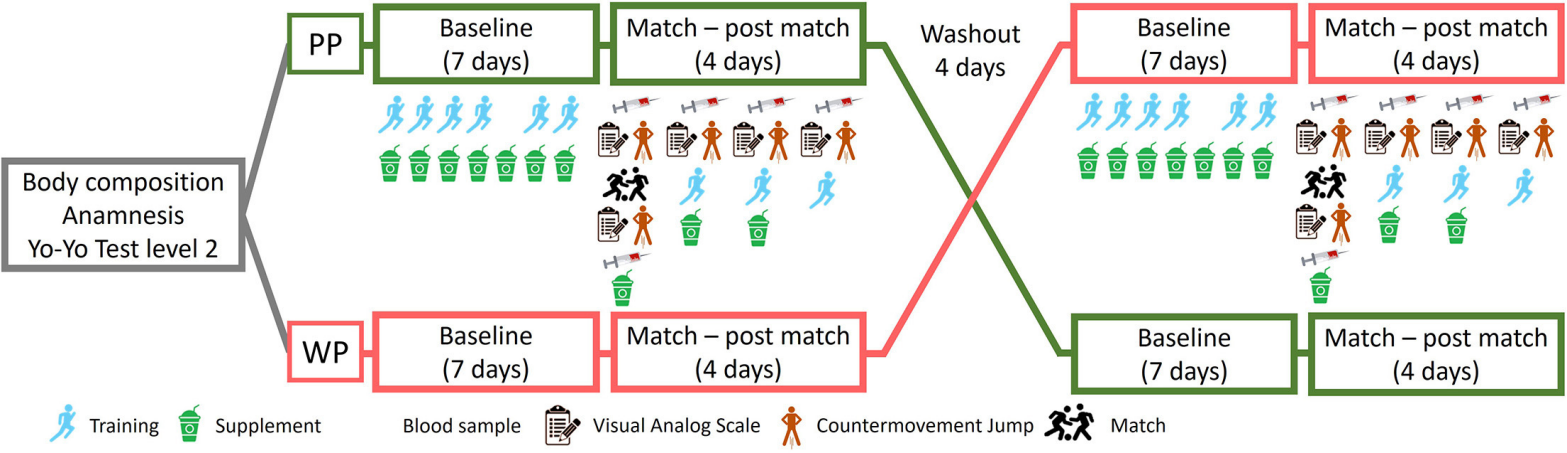
Crossover study design with whey protein (WP) supplementation or pea protein isolate (PP). The groups received supplementation for 10 continuous days. After 4 days of washout, the groups were reversed.

This study used a double-blind, randomized crossover design with a Brazilian soccer team, as shown in Figure 1. During the first week, body composition was assessed using dual-energy X-ray absorptiometry. The participants were then interviewed by a nutritionist to conduct an anamnesis with questions regarding habitual food intake, consumption of supplements, intestinal function, history of injuries, allergies, intolerance, and personal data.

The participants were stratified based on their positions in the field to ensure an equitable distribution of roles across the supplementation groups. Following this stratification, athletes were randomly allocated to the PP (*n* = 6) and WP groups (*n* = 6) using Randomizer software (Medical University of Graz, Graz, Austria). As a result, each group comprised athletes from various positions, thereby minimizing the potential impact of position-specific physical demands on the study outcomes. The athletes received the investigational products in a blinded manner for ten consecutive days, always immediately after training. On the 7th day of supplementation, they were subjected to a soccer game. The athletes received supplementation for 3 days after the game (the 10th day of the first study phase).

The game consisted of two 45-min periods with a 15-min (halftime) rest interval between them. On the day of the game, blood samples, pain scale tests, and countermovement jumps were obtained before (pre) and immediately after the game (post). The same tests and blood collections were repeated on the following 3 days (24 h, 48 h, and 72 h), always before the beginning of the training sessions. After a washout period of 4 days, the groups were reversed (Figure 1).

### Participants

**Figure 2:**
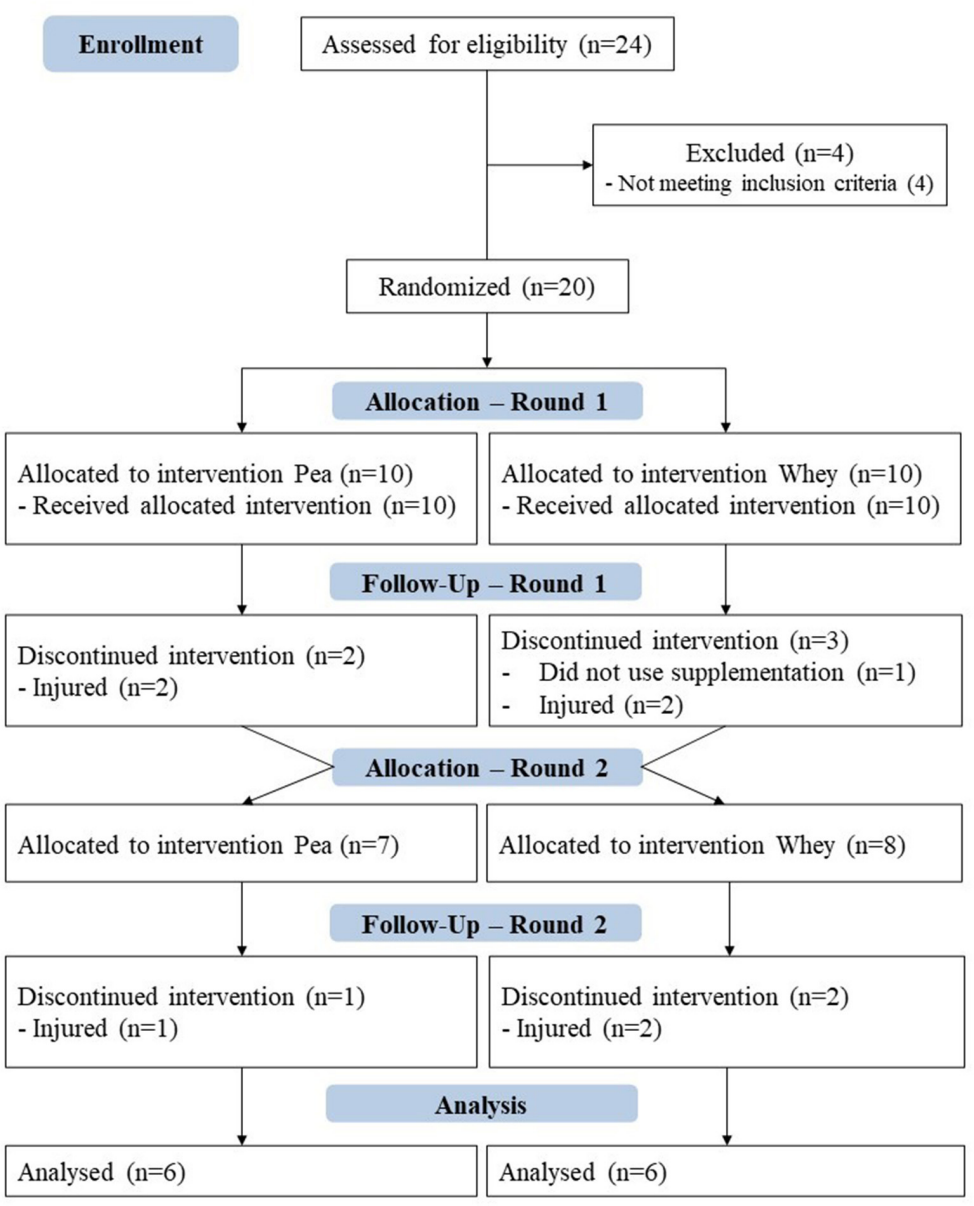
CONSORT diagram of the data collection process.

Athletes who trained at least 2 h daily, 6 days a week, and have participated in national competitions for at least 2 years were selected. Goalkeepers or athletes who did not participate in all collections had injuries, or used nutritional supplements, stimulants, sleep or appetite suppressants, analgesics, or any other substance that interfered with metabolism were excluded from the study. Of the 24 athletes on the team, four were excluded for not meeting the inclusion criteria. Thus, the intervention started with ten athletes in each group, each athlete representing a different soccer position. By the end of the study, eight athletes were excluded either due to injuries or non-compliance with the supplementation protocol (Figure 2).

The study was conducted following the Declaration of Helsinki, and ethical clearance was obtained from the University Hospital of the Federal University of Rio de Janeiro (protocol number: 64210322.0.0000.5257). The study was registered in The Brazilian Registry of Clinical Trials under protocol number 13478. All the participants provided written informed consent before undergoing the procedure.

### Supplementation

The volunteers received powder supplementation containing 0.5 (g/k) of protein from PP (Cosucra, Peqc, Belgium) or WP (Nutrata, SC, Brazil) and 0.02 g/kg of lipid. Maltodextrin (Athletica Nutrition, SP, Brazil) was added until 0.25 g/kg of carbohydrate (CHO) was reached. Aminograms were determined using high-performance liquid chromatography (34) (Supplementary Table 1). Supplementation was prepared at the training site and delivered to the athlete after training in a single dose diluted in a non-transparent bottle containing 350 mL of water.

### Assessment of anthropometric and body composition

A Filizola scale with a stadiometer was used to assess body mass and height. Body fat percentage, bone mass, fat-free mass, and fat tissue mass were assessed through Healthcare dual-energy X-ray absorptiometry with Encore 2008 version 12.30 software. Prior to the assessment, the athletes fasted for 4 h for solids and 2 h for liquids. In the last 24 h before the examination, they refrained from engaging in any physical activity and did not use any metal objects during the examination. The DXA equipment was calibrated before the evaluations and a phantom scan was performed to confirm the results.

### Food intake

The dietary intake of each athlete was assessed using a 24-h recall collected by a nutritionist. For each athlete, three 24-h food recalls were collected weekly throughout the study period: two on weekdays and one on weekends. Quantitative analysis of energy and macronutrient intake at each meal was performed using the USDA Food Composition Table (version 28) (35). Additional nutritional information was obtained from food labels when not present in the food composition table.

### Blood collection

Serum (BD SST^®^ II Advance^®^ Vacutainer 8.5 mL tube) and plasma (BD Vacutainer^®^ Plus with sodium heparin 10 mL tube) samples were collected and maintained on ice for transport. Plasma and serum were separated by centrifugation (1500 g for 20 min at 4 °C) and stored at −80 °C. Blood collections were performed in fasting conditions before the athletes had breakfast at the club, both pre-game and at 24 h, 48 h, and 72 h after the game. Post-game collections occurred 5–8 min after the end of the game and before supplementation.

### Biochemical analysis

Creatine kinase (CK), aspartate transaminase (AST), alanine transaminase (ALT), lactate (LA), urea, creatinine, and uric acid levels were analyzed according to the manufacturer's instructions by using commercial kits (RANDOX) on a semiautomatic biochemical analyzer (Daytona RX, Randox). All measurements were performed in duplicates.

### NMR spectroscopy

The use of metabolomics to assess the interactions between nutrition and exercise is increasing, and metabolomics can be used to monitor changes in the physiological state of soccer athletes (32, 36). In addition, a better understanding of metabolic changes can help assess the functioning of nutritional strategies to improve recovery (37).

All NMR experiments were performed as described by Oliveira et al. (38) using a 500 MHz Avance DRX spectrometer (Bruker Biospin, Karlsruhe, Germany) at 298 K. One-dimensional 1H NMR spectra were obtained using a standard 1 D CPMG pulse sequence to suppress signals from macromolecules through a T2 filter (84 ms) using 1024 scans. All spectra were referenced to the chemical shift of the anomeric proton signal of α-glucose at δ 5.22 ppm, and edge effects were evaluated by overlaying all spectra using TopSpin 3.2 (Bruker Biospin, Rheinstetten, Germany). Two-dimensional (2D) 1H−1H TOCSY spectra were acquired with acquisition parameters of 4096 × 512 points for selected samples to confirm metabolite assignment. The spectral width was 12,934 Hz, with a relaxation delay of 3 s and a spin-lock time of 60 ms.

The assignment of resonances for the whole spectrum was carried out following the literature (39), using the Human Metabolome Database (http://www.hmdb.ca/) (40), with an in-house database of one- and two-dimensional NMR spectra of reference compounds, and two-dimensional TOCSY experiments confirmed it.

### Countermovement jump

The countermovement jump (CMJ) test is a well-accepted method for assessing lower body power, a crucial physical attribute in soccer. Fatigue and insufficient recovery often decrease in CMJ performance, making it a sensitive and valuable measure of an athlete's readiness to perform (41, 42). The CMJ was performed on a force plate (Jump System Pro-Cefise, Brazil), according to Lombard et al. (43), at pre- and post-game moments and 24 h, 48 h, and 72 h. At 24 h, 48 h, and 72 h, jumps were performed before training.

Initially, the 30 m sprint test, a recognized method for fatigue assessment (41), was considered for the study. However, during round 1, it was noted that this test posed a significant injury risk to participants. Regrettably, injuries from this test sidelined four athletes, leading to the discontinuation of its use for the safety of the participants.

### Visual analog scale

The athletes individually assessed their perception of pain using a visual analog scale (VAS) of 1–10 points (44) at pre- and post-game moments and 24 h, 48 h, and 72 h.

### Yo-yo intermittent recovery test level 2

Yo-Yo intermittent recovery test level 2 (YYIRT) was conducted at the beginning of the study according to established procedures, which have been described in the literature (45) for the characterization of the physical conditioning of athletes. The test was used to exclude baseline differences in the physical performance level.

### Movement patterns during the game

During the entire game period, displacement in the field (km), average speed (km/h), maximum speed (km/h), distance covered with speed above 15 km/h (m), and heart rate (beats/min) of the athletes were collected using GPS and a heart rate monitor (Polar RS400).

### Statistical analysis

The number of participants in the study was determined using convenience sampling, given the impossibility of select samples through other ways and the difficulty to achieve soccer players to experimental procedures. The statistical power (1-β) of 0.84 was calculated in GPower software (version 3.1.9.6, Universität Kiel, Germany) based on partial η^2^ of time in increase of CK levels (0.11), alpha = 0.05 and *n* = 12.

Statistical analysis was conducted using SPSS software (version 20.0; SPSS Inc., Chicago, IL, USA) and Metaboanalyst 4.0 for NMR spectral analysis. For parametric data, results are presented as mean ± standard deviation (SD). Non-parametric data are presented as median, with minimum and maximum values. The level of significance was set at α = 0.05.

The distribution of data was assessed using the Shapiro–Wilk test. To identify baseline differences between groups and differences in area under curve between groups, we employed the paired samples t-test for parametric data, or the Mann-Whitney test for non-parametric data. We also performed Cohen's d to evaluate the effect size of supplementation on AUC. The values of Cohen's d for small, medium and large effects were 0.2, 0.5 and 0.8, respectively.

Efficacy endpoints were assessed using a two-way repeated-measures analysis of variance (RM ANOVA) for parametric data. The model included treatment period (PP vs. WP) and time (pre- and post-game, and at 24 h, 48 h, and 72 h after the game) as within-subject factors. Sidak's *post-hoc* test was used to compare groups (WP × PP) at the same time and times within each group. The magnitude of changes in biochemical parameters were evaluated using effect size analysis (partial η^2^), to mitigate potential type II errors given the small sample size of the study (*n* = 12). The values of partial η^2^ for small, medium and large effects were 0.01; 0.06 and 0.16 respectively (46).

Each NMR spectrum was analyzed by integrating the bucket size regions of 0.03 ppm using AMIX software (Bruker Biospin, Rheinstetten, Germany). The bucket tables were normalized using the sum of intensities, and the data were subjected to the Pareto scaling method (47) using AMIX software.

Partial Least Squares Discriminant Analysis (PLS-DA) and Variable Importance in Projection (VIP) scores were obtained for each model using Metaboanalyst 5.0. These helped to identify the most significant metabolic differences between groups.

In addition to the metabolites considered relevant in terms of VIP scores, we subjected all spectral regions (buckets) and biomarkers to a univariate two-way repeated-measures ANOVA analysis for parametric data. Notably, PLS-DA is a multivariate analysis technique used to maximize variation between different sample groups, while VIP scores are used to indicate the importance of each variable in the PLS-DA model projection.

## Results

Twelve male athletes aged 18.4 ± 0.7 years with a body mass of 69.4 ± 6.5 kg, height of 1.76 ± 0.06 m, body mass index of 22.3 ± 1.7 kg/m^2^, and body fat of 16.26 ± 4.65% participated in the study. Protein powder differed only in the number of carbohydrates, arginine, cysteine, and glycine (*p* < 0.05) (Supplementary Table 1). No significant differences were found between the groups in energy or macronutrient intake (Table 1).

**Table 1:** Values of dietary intake assessed by a 24-h dietary record.

	**WP**	**PP**	***p*-value**
Energy (kcal)	3477.68 ± 1370.33	3332.91 ± 964.97	0.791
Protein (g/kg)	2.35 ± 0.81	2.26 ± 0.83	0.666
Proteín (% kcal)	19.05 ± 2.82	18.68 ± 4.53	0.391
Charbohydrate (g/kg)	7.01 ± 2.49	7.12 ± 2.88	0.845
Charbohydrate (% kcal)	56.7 ± 8.88	57.71 ± 11.79	0.758
Lipids (g/kg)	1.41 ± 0.82	1.38 ± 1.11	0.933
Lipids (% kcal)	24.25 ± 8.71	23.6 ± 9.73	0.942

PP, pea protein; WP, whey protein. The results were presented as mean ± standard deviation. There was no statistical difference between the groups (*p* > 0.05).

**Figure 3:**
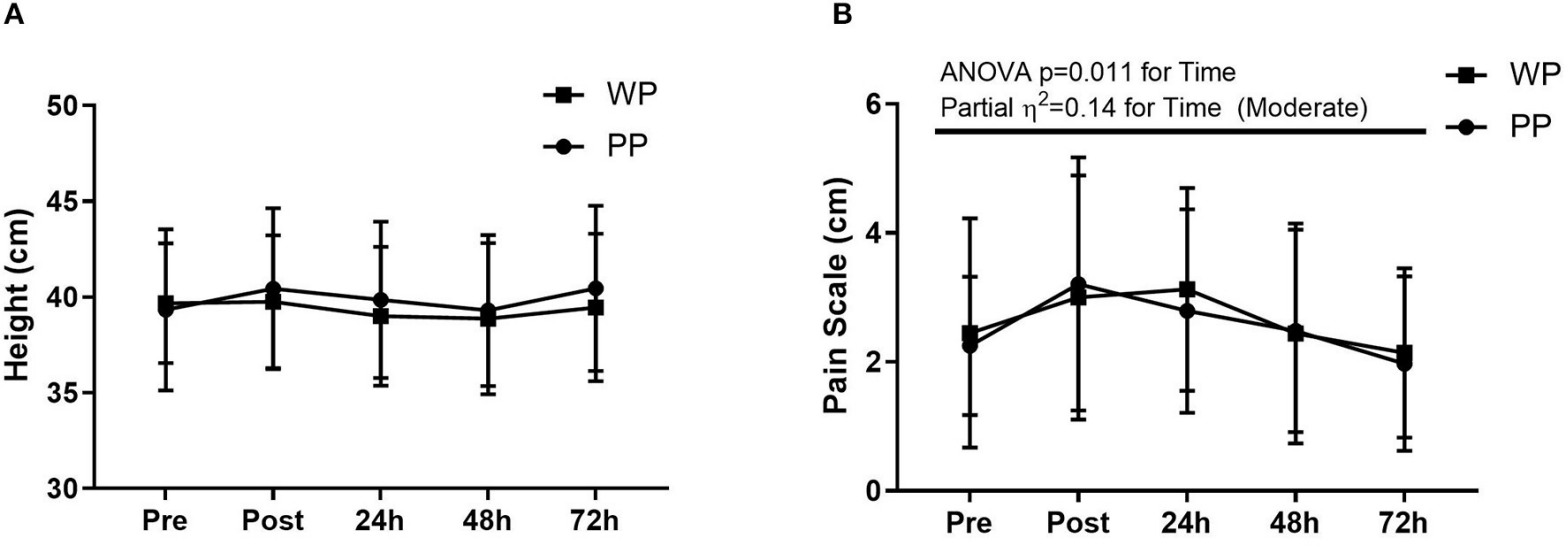
Countermovement jump **(A)** and pain scale **(B)** pre match (Pre), post match (Post), 24 h, 48 h, and 72 h after match.

There was no difference in YYIRT, distance covered in the field, average speed, maximum speed, and distance traveled at speeds above 15 km/h between the game played in the first and second rounds (*p* > 0.05) (Table 2). There are also no significant differences between the groups and within groups on CMJ or VAS, with a moderate impact of supplementation on CMJ (partial η^2^ = 0.06) and a moderate impact of time (partial η^2^ = 0.14) on the VAS (Figure 3).

**Table 2:** Data evaluated in YYIRT2 and movement patterns during the two games, presented in median, minimum, and maximum (*n* = 12).

	**Median**	**Minimum**	**Maximum**
**YYIRT2**			
Completed stages	24	15	30
Distance covered (m)	960	600	1,200
**Game (Round 1)**			
Distance covered (m)	7,650	6,600	9,800
Average speed (km/h)	7.9	6.2	9.6
Maximum speed (km/h)	29.5	26.2	34.5
Distance covered >15 km/h (m)	1,360	790	1,850
**Game (Round 2)**			
Distance covered (m)	7,500	6,000	9,350
Average speed (km/h)	8.1	5.9	9.8
Maximum speed (km/h)	29	24.5	35.2
Distance covered >15 km/h (m)	1,250	800	1,750

YYIRT2, Yo-Yo intermittent recovery test level 2.

**Figure 4:**
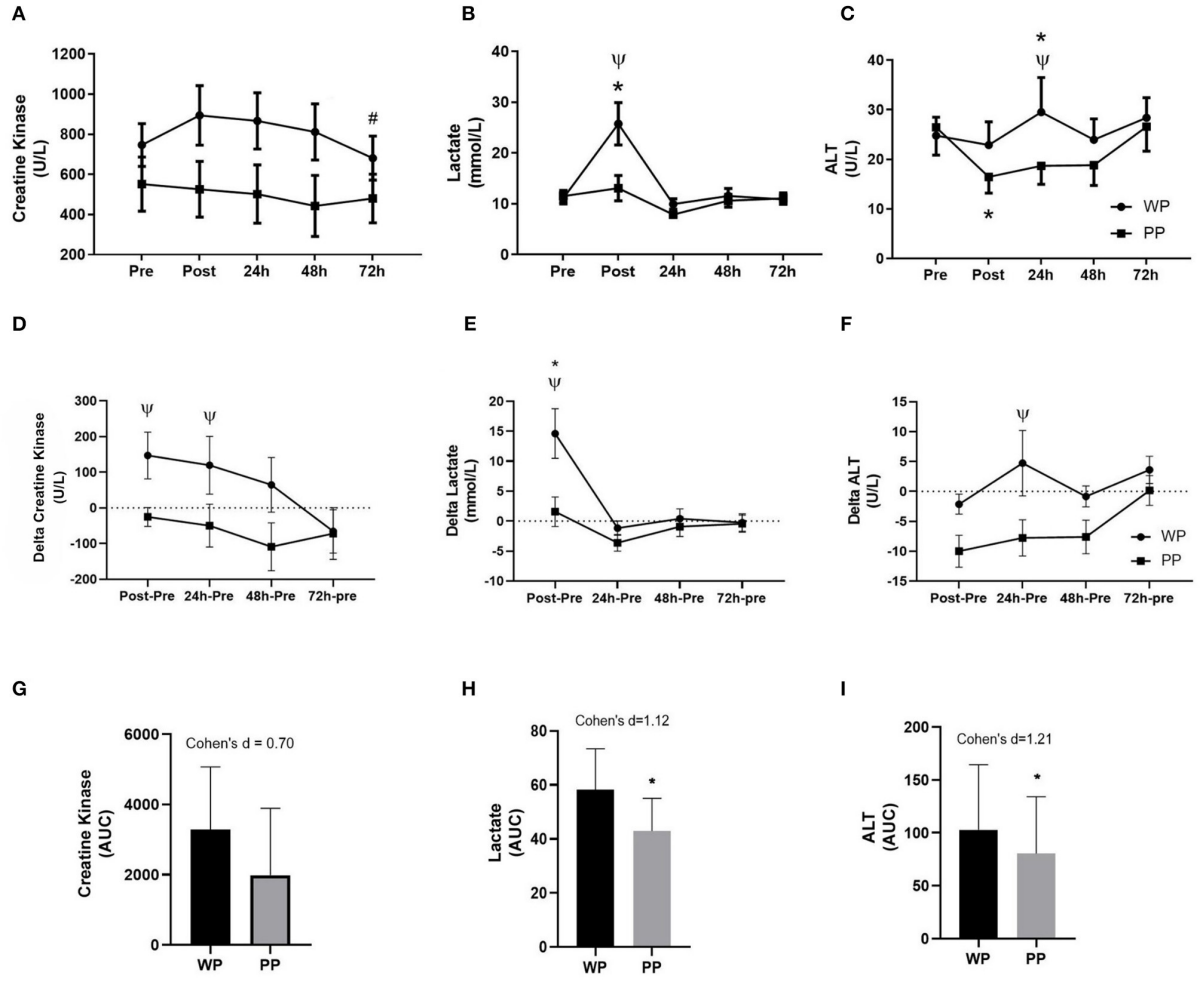
Kinetic behavior of biochemical markers during the experimental timeline Creatine Kinase **(A)**; Lactate **(B)** and Alanine Aminotransferase (ALT) **(C)**. Delta values of Creatine Kinase **(D)**, Lactate **(E)**, and ALT **(F)** compared to pre-game values. ^#^Significant difference with the post-game in the WP group. *Significant difference between moments within groups (*p* < 0.05). ^*Ψ*^DIFFERENCE between groups at the same time (*p* < 0.05). Area under curve of Creatine Kinase **(G)**, Lactate **(H)**, and ALT **(I)**. *Significant difference between groups (*p* < 0.05).

Comparing the values of the biochemical markers between the supplemented, LA differed in the post-game. Regarding within-group assessment, in the WP group, LA differed between pre- and post-game moments, and urea varied between pre-game moments and 24 h. On the other hand, in the PP group, only ALT differed between pre- and post-game moments (Table 3) (Figures 4A–C). The area under the curve (AUC) was measured for CK, LA, and AST kinetics over time. The effect size of supplementation was medium in CK (Cohen's d = 0.7) and large in LA (Cohen's d = 1.12) and AST (Cohen's d = 1.21) (Figures 4G–I).

**Table 3:** Absolute values of biochemical markers for each time of collection in the groups (mean ± standard deviation).

	**CK**	**AST**	**ALT**	**LA**	**Cr**	**Urea**	**UA**
**WP**							
Pre-game	747.4 ± 369.5	28.1 ± 7.4	24.8 ± 12.6	11.1 ± 4	0.8 ± 0.1	22.6 ± 6.3	3.5 ± 0.8
Post-game	894.5 ± 513.6	28.4 ± 11.1	22.9 ± 16.2	25.8 ± 14.4^¥*^	0.8 ± 0.2	22.2 ± 5.8	3.6 ± 0.6
24 h	867.2 ± 486.1	25.1 ± 10	29.5 ± 24.1	10 ± 3.5	0.7 ± 0.1	17.4 ± 7.6^¥^	3.2 ± 0.7
48 h	812.2 ± 483.9	25.6 ± 9	23.9 ± 14.6	11.5 ± 5.1	0.7 ± 0.2	21.9 ± 4.8	3.5 ± 0.5
	681.7 ± 380.6^$#^	25.1 ± 7.5	28.4 ± 14.1	10.9 ± 3.4^#^	0.8 ± 0.1	22.1 ± 5	3.8 ± 0.6^#^
**PP**							
Pre-game	552 ± 466.6	26.1 ± 10	26.4 ± 19.3	11.5 ± 4	0.7 ± 0.2	22 ± 4.7	4 ± 1.1
Post-game	526.6 ± 480.8	22.9 ± 10.6	16.4 ± 11.2^¥^	13.1 ± 8.6	0.7 ± 0.2	19.7 ± 7	3.7 ± 1.1
24 h	502.4 ± 503.1	20.5 ± 8.2	18.7 ± 12.9	7.9 ± 2.5	0.7 ± 0.2	21.3 ± 6.1	3.5 ± 0.9
48 h	443 ± 527.1	21.6 ± 16.6	18.8 ± 14.2	10.6 ± 4.4	0.7 ± 0.1	21.1 ± 4.7	3.5 ± 0.8
72 h	480.1 ± 419	22.6 ± 8.6	26.6 ± 17.1^#^	11.1 ± 3.7	0.8 ± 0.2	23.7 ± 4.4	4 ± 0.8
Partial η^2^ Protein	0.58 (large)	0.15 (moderate)	0.10 (moderate)	0.07 (moderate)	0.03 (small)	0.00 (none)	0.05 (small)
Partial η^2^ Time	0.11 (moderate)	0.10 (moderate)	0.15 (moderate)	0.32 (large)	0.12 (moderate)	0.13 (moderate)	0.15 (moderate)
Partial η^2^ Interaction (time × protein)	0.10 (moderate)	0.02 (small)	0.09 (moderate)	0.17 (large)	0.03 (small)	0.10 (moderate)	0.03 (small)

CK, Creatine kinase; AST, aspartate transaminase; ALT, alanine transaminase; LA, lactate; CR, Creatinine; UA, Uric Acid; WP, whey protein; PP, pea protein. The results were presented as mean ± standard deviation. ^*^Significant difference between groups (*p* < 0.05), ^¥^Significant difference between pre- and post-game moments (*p* < 0.05). ^$^Significant difference compared to post-game moment within group (*p* < 0.05). ^#^Significant difference compared to 24 h moment within group (*p* < 0.05).

Evaluating the delta values (value of a given moments minus the value of the pre) between the groups, CK presented lower values in PP group in the post-game moment, 24 h, and 48 h compared to WP group (partial η^2^ = 0.58 for protein type supplementation), ALT decrease in the post-game moment and 72 h in PP group, with no statistical differences between groups (partial η^2^ = 0.24 for protein type supplementation), and LA was lower in PP group, in the post-game moment (partial η^2^ = 0.12 for protein type supplementation) (Figures 4D–F).

The PLS-DA score graph (paired comparison) showed poor differentiation between groups at all time points (Table 4). In the permutation test (*n* = 1000), pre- (*p* = 0.835) and post-game moments (*p* = 0.408) and at 24 h (*p* = 0.727), 48 h (*p* = 0.951), and 72 h (*p* = 0.959) did not show any significant difference.

**Table 4:** PLS-DA cross-validation between the WP and PP in pre-game, post-game, 24, 48, and 72h after game moments.

	**Measure**	**1 comps**	**2 comps**	**3 comps**	**4 comps**	**5 comps**
Pre	Accuracy	0	0.042	0.083	0.083	0.042
	R2	0.23	0.305	0.523	0.668	0.74
	Q2	−1.392	−1.683	−1.808	−2.19	−2.22
Post	Accuracy	0.667	0.583	0.417	0.333	0.333
	R2	0.209	0.334	0.414	0.733	0.86
	Q2	−0.009	−0.107	−0.569	−1.472	−1.517
24h	Accuracy	0.208	0.292	0.25	0.208	0.208
	R2	0.221	0.275	0.426	0.554	0.674
	Q2	−0.947	−1.054	−1.444	−2.965	−3.414
48h	Accuracy	0.375	0.292	0.375	0.333	0.5
	R2	0.092	0.347	0.649	0.698	0.745
	Q2	−0.226	−0.811	−0.773	−0.814	−0.612
72h	Accuracy	0.333	0.125	0.25	0.292	0.208
	R2	0.079	0.367	0.429	0.514	0.631
	Q2	−0.183	−1.257	−1.147	−1.319	−2.276

**Figure 5:**
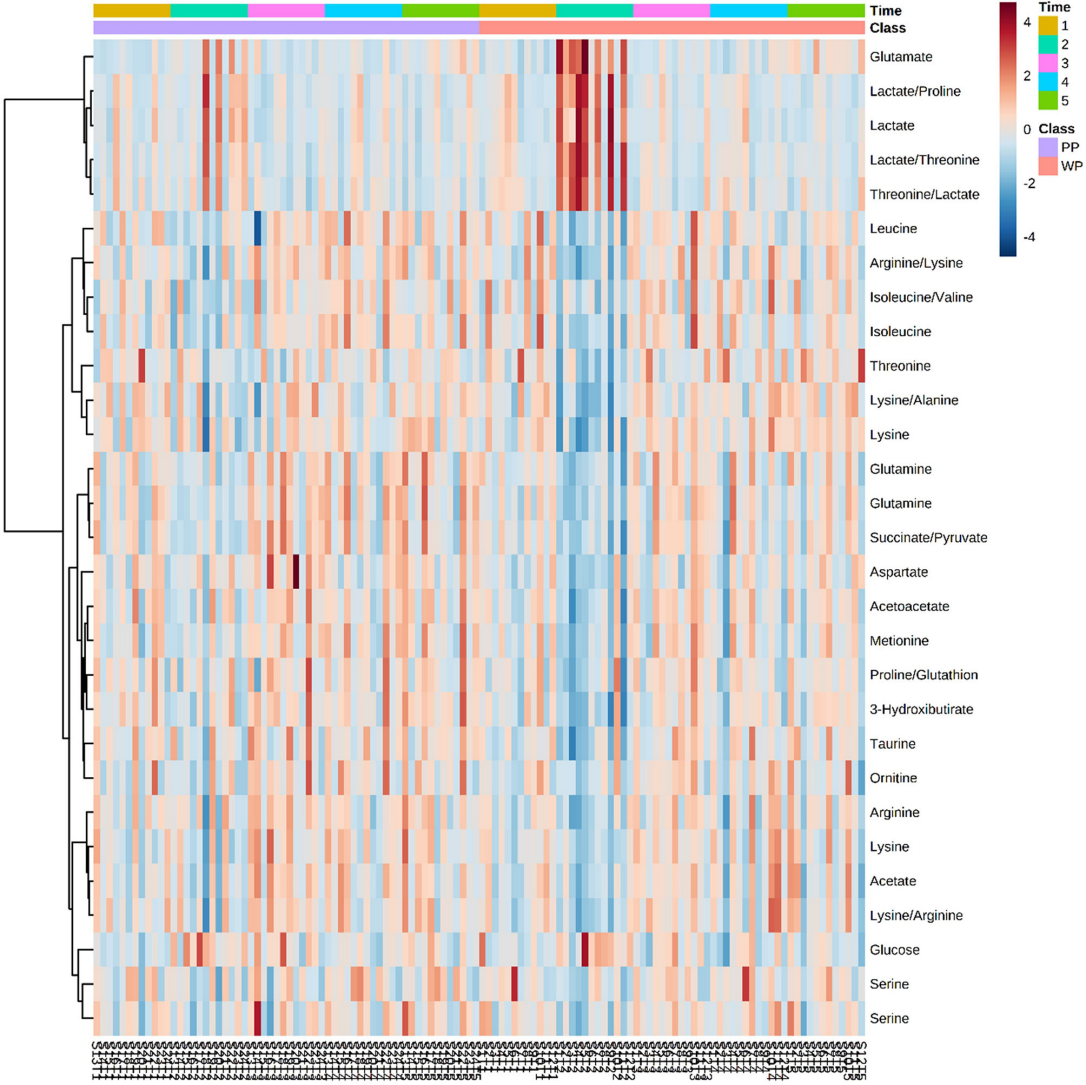
Heatmap with metabolites that have at least one association with a *P*-value < 0.05 in Anova 2-way test 1 – Pre match, 2 – Post match, 3 – 24 h, 4 – 48 h, and 5 – 72 h. Each column represents an athlete at a given time of collection.

Although no significant differences were found between the groups using the PLS-DA method, soccer games promoted changes in metabolites between groups and moments in each protein supplementation group. Significant differences were observed in 22 of the 48 identified metabolites, including amino acids, ketone bodies, tricarboxylic acid cycle metabolites, and glucose metabolism (Figure 5). Glutamate and LA levels significantly increased between pre- and post-game moments in the WP group, causing a difference between the groups when the post-game moment was evaluated. By contrast, several metabolites, such as taurine, serine, betaine, ketoisolaverate, and proline, were reduced in the WP group after the game; such condition was not observed in the PP group. However, when comparing the groups, no difference was found in metabolites in the pre-game moment and at 48 h and 72 h (Table 5).

**Table 5:** Metabolites associated with WP and PP by time or group (*p*-value).

	**WP**				**PP**				**WP** × **PP**				
	**Post**	**24 h**	**48 h**	**72 h**	**Post**	**24 h**	**48 h**	**72 h**	**Pre**	**Post**	**24 h**	**48 h**	**72 h**
3-Hydroxibutirate	0.000^*^	0.689	0.486	0.581	0.044^*^	0.598	0.828	0.230	0.340	0.008^¥^	0.280	0.155	0.111
Acetoacetate	0.005^*^	0.314	0.567	0.782	0.080	0.602	0.650	0.531	0.480	0.071	0.827	0.410	0.293
Arginine	0.015^*^	0.278	0.824	0.523	0.320	0.151	0.784	0.468	0.558	0.04^¥^	0.345	0.928	0.501
Arginine / Lysine	0.001^*^	0.621	0.865	0.629	0.084	0.147	0.144	0.445	0.570	0.191	0.688	0.284	0.497
Aspartate / Methylguanidine	0.064	0.693	0.696	0.439	0.760	0.011^*^	0.641	0.686	0.459	0.024^¥^	0.004^¥^	0.506	0.711
Glucose	0.000^*^	0.891	0.298	0.323	0.000^*^	0.004^*^	0.342	0.041^*^	0.107	0.283	0.224	0.716	0.150
Glutamate	0.000^*^	0.635	0.645	0.05^*^	0.018^*^	0.756	0.701	0.129	0.978	0.001^¥^	0.848	0.917	0.621
Glutamine	0.000^*^	0.269	0.556	0.881	0.013^*^	0.190	0.614	0.149	0.677	0.361	0.551	0.051	0.292
Isoleucine	0.000^*^	0.997	0.594	0.269	0.037^*^	0.827	0.057	0.542	0.305	0.516	0.416	0.153	0.489
Isoleucine/Valine	0.006^*^	0.786	0.706	0.349	0.018^*^	0.997	0.127	0.941	0.362	0.647	0.523	0.803	0.922
Lactate	0.000^*^	0.854	0.980	0.616	0.239	0.437	0.895	0.533	0.995	0.000^¥^	0.547	0.870	0.897
Leucine	0.002^*^	0.724	0.831	0.397	0.064	0.801	0.537	0.224	0.308	0.673	0.675	0.847	0.296
Lysine	0.000^*^	0.740	0.555	0.296	0.033^*^	0.369	0.283	0.931	0.804	0.024^¥^	0.327	0.159	0.475
Lysine / Alanine	0.001^*^	0.750	0.785	0.401	0.013^*^	0.407	0.262	0.809	0.669	0.120	0.932	0.333	0.512
Lysine / Arginine	0.036^*^	0.308	0.274	0.730	0.149	0.295	0.918	0.579	0.614	0.233	0.595	0.623	0.476
Ornitine	0.361	0.379	0.724	0.836	0.746	0.002^*^	0.412	0.219	0.782	0.752	0.036^¥^	0.371	0.452
Proline / Glutathione disulphide	0.006^*^	0.157	0.385	0.777	0.144	0.672	0.864	0.631	0.450	0.038^¥^	0.801	0.077	0.343
Serine	0.007^*^	0.470	0.682	0.527	0.048^*^	0.489	0.402	0.736	0.375	0.905	0.391	0.646	0.937
Succinate / Pyruvate	0.002^*^	0.367	0.457	0.892	0.005^*^	0.036^*^	0.476	0.470	0.787	0.494	0.134	0.088	0.393
Taurine / Glucose	0.005^*^	0.212	0.481	0.377	0.156	0.616	0.696	0.573	0.325	0.017^¥^	0.815	0.501	0.506
Threonine	0.000^*^	0.412	0.757	0.414	0.068	0.143	0.183	0.400	0.991	0.053	0.503	0.101	0.099

PP, pea protein; WP, whey protein. ^*^Significant difference compared to the pre-match moment in the WP or PP groups (*p* < 0.05). ^¥^Significant difference between the WP and PP groups (*p* < 0.05).

**Figure 6:**
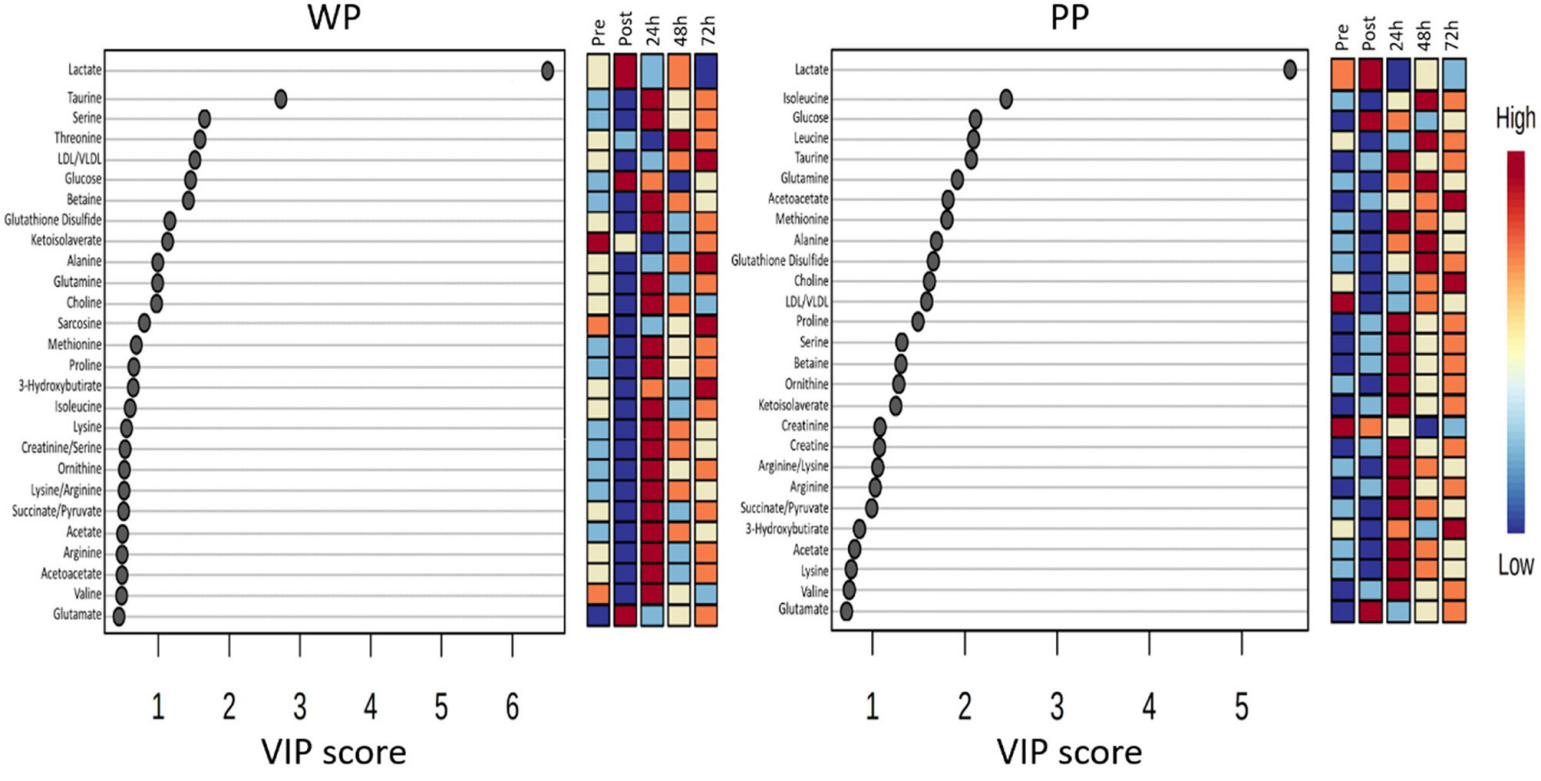
The most significant metabolites identified in the PLS-DA model were plotted with their VIP score in whey protein (WP) and pea protein (PP) groups in pre, post, 24 h, 48 h, and 72 h moments.

The VIP score analysis plot displays the 27 metabolites with the highest scores (Figure 6). Certain metabolites were only identified in one of the groups, such as Threonine, Betaine, Sarcosine, and 3-Hydroxybutirate in the WP group, and Leucine and Creatine in the PP group. Among the top 10 metabolites with the highest VIP scores, Lactate was the only metabolite that ranked the same in both groups, appearing as the metabolite with the highest score.

The VIP method allows us to observe metabolic alterations at each time point in the study. Upon examining the temporal behavior of the metabolites, we noted that taurine decreased immediately after the game in the WP group but increased 24 h post-game and returned to baseline 48 h post-game in both groups. In contrast, several other metabolites displayed similar trends between the groups, including Methionine, Ornithine, Acetate, Lysine, and Glutamate.

## Discussion

This study assessed whether type of protein source influences biochemical and metabolic outcomes after a soccer game. In our findings, CK delta values were different between groups in Post-Pre, 24 h-Pre and 48 h-Pre moments and LA differed between groups at post-game with lower levels of PP compared to WP supplementation, with a high impact of supplementation on this difference. Of the 48 metabolites analyzed, 22 had changes when compared to the pre-game moment. Glutamate increased, while taurine, serine, betaine, ketoisolaverate, and proline were reduced in the WP group after the game; such condition was not observed in the PP group.

Sarcosine, an intermediate product in the glycine metabolic pathway, increased significantly at 72 h after the game in the WP group. Spectral analysis using NMR of serum from modern pentathlon athletes during a three-week training period demonstrated an incremental rise in sarcosine levels week after week. This suggests an accumulative effect of this marker in response to the stress imposed by the progressive training weeks (48).

Felder et al. (49) reported that increased sarcosine levels after exercise (a 10-week program) may be related to oxidative stress. Sarcosine can be formed by the serine-glycine-sarcosine or choline-betaine-dimethylglycine-sarcosine metabolic pathways. According to the VIP scores, serine, betaine, and choline levels decreased after exercise in the WP group. Feder et al. (49) cited the role of glutamate in the metabolic pathway of sarcosine and reported its reduction at 48 h after exercise. In addition, we observed an increase in glutamate levels in both groups, which may be related to hemolysis generated by exercise (50).

A study that investigated the effect of a supplement containing serine, valine, and arginine found a lower level of fatigue during exercise and a lower reduction of these amino acids after exercise compared with the placebo group (51). In addition to valine, isoleucine, and leucine (BCAAs) also decrease after exercise (50, 52, 53) and return after recovery (54).

Our study showed reduced in BCAAs and ornithine in both supplemented groups after a soccer game. The catabolism of these amino acids plays a fundamental role in protein synthesis, in which they can be oxidized in skeletal muscle and energy production (53). The diminution of these metabolites could suggest an auxiliary pathway for energy production during exercise, especially amidst intense or sustained efforts such as those found in a soccer match, which could concurrently explain the observed alteration in ketone bodies (55).

Ketone bodies can be synthesized from amino acids, such as leucine, lysine, valine, or glycine, which explains the reduction of these amino acids after exercise (56). The concentration of ketone bodies and their precursors increases in different body fluids (56). We found different results when comparing the two groups. In both groups, 3-hydroxybutyrate, acetoacetate, and acetate levels immediately decreased after exercise. However, according to the VIP score, the WP group had a maximum peak 24 h after the game and the PP group only after 72 h.

Taurine plays an important role in the contractile function of skeletal muscles and helps in the antioxidant defense in response to stress (57). Pitti et al. (32) also observed decreased salivary taurine levels in young soccer players after matches. Marinho et al. (58) found reduced values of urinary taurine after the game in Brazilian U-20 soccer players and associated this reduction with muscle tissue damage due to sprints, accelerations, and jumps performed during the game. Ra et al. (36) suggested that the significant increase in taurine 3 days after a soccer match may be related to fatigue markers.

The VIP score and heatmap data showed that taurine reduced immediately after the game (WP group), increased 24h after the game, and normalized 48 h after the game in both groups. Despite the similar behavior, an increase in taurine 24 h after the game was observed to be higher in the WP group than in the PP group.

The post-exercise lactate concentrations detected in our study (biochemical and NMR analysis) potentially provide a window into the complex interplay of active energy systems during a soccer game (59). The varied motor skills demonstrated by soccer players, each demanding distinct energy delivery, might result in different lactate responses post-game. It is possible that this finding is related to the sprints and distances covered by the WP group athletes in the games, without being related to more significant cell damage.

Although previous studies reported an increase in CK, AST, and ALT up to 48–72 h after a professional soccer game (3, 60), our data do not corroborate these findings. Protein supplementation might have attenuated the increase in CK levels after the game. Several studies have assessed the effects of post-exercise protein supplementation. However, few studies have focused on muscle recovery and reduction of cell damage (17). A systematic review indicated that animal protein supplementation can reduce the rise in CK levels by up to 40% (18).

Other studies have also demonstrated the beneficial effects of protein supplementation in reducing muscle damage after high-intensity exercise (61–63). In our study, absolute delta values were lower in PP group compared to WP group, with large effect. ALT and AST are related to liver function, but these biomarkers can be found in skeletal muscle tissue (64). The effect of protein supplementation for these biomarkers was moderate and they presented no modification in their kinetics in 72 h after the game. All these data suggest that short-term PP can reduce muscle damage, corroborating with the literature.

In a study conducted by Huang et al. (61), marathon athletes received WP or CHO supplementation for 5 weeks. The WP group had lower CK, AST, and ALT levels on the day after the race and 1 week later. The increase in muscle damage can be related to pain and loss of muscle power, but although a difference between groups in CK levels was noted (a biomarker of muscle damage), no significant differences in perception of pain and muscle power performance (measured by CMJ) were observed during evaluations. The increase of CK during experimental design was compatible with those already found before (65). Thus, the absence of differences between groups on perception of pain and muscle power can be explained by an expected (and normal) increase in muscle damage after a match.

To assess whether vegetable protein would benefit the recovery of athletes, Kritikos et al. (66) investigated the impact of WP and Soy Protein supplementation in soccer players up to 48 h after the physical test. Their findings showed no difference between groups for CK and muscle pain (for all time points), but protein carbonyl after 48 h of the test was lower in the Soy group when compared to placebo. Shenoy et al. (63) evaluated the effect of soy protein supplementation for four weeks in a training protocol that induced muscle damage in trained volunteers. The supplemented group had lower CK and C-reactive protein levels than the placebo group.

Compared with soy protein, PP is characterized by its high digestibility and has relatively less allergenic responses (67). In addition, PP is a relatively new type of vegetable protein. It is becoming increasingly popular in the food industry because of its availability, low cost, nutritional value, and health benefits (23).

Due to the nutritional quality of PP, Babault et al. (68) compared the effects of daily supplementation with WP, PP, and placebo after 12 weeks of strength training. They observed no difference between the protein sources for biceps brachii muscle thickness, 1-RM maximal strength, isometric, concentric, and eccentric maximal torque when the amounts of amino acids were similar. Banaszek et al. (20) conducted the only comparative study on protein sources to assess muscle recovery in CrossFit athletes. When supplemented for 8 weeks, they found no differences in strength, performance, or body composition compared with WP.

Other plant sources have also been shown to reduce muscle damage. For example, downhill runners received oat-based protein or CHO supplementation 14 days before a race and 4 days later. The oat-based protein group showed a reduced elevation of muscle damage and inflammatory markers (CK, C-reactive protein, myoglobin, and IL-6). The authors argued that the effect of supplementation could be explained by the combination of anti-inflammatory and antioxidant factors present in oat proteins (62). Although the polyphenol content (69) may have influenced the results (70), no studies evaluated the anti-inflammatory and antioxidant effects of PP in athletes.

Evaluating the total distance covered, distance covered above 15 km/h, and maximum speed, the athletes performed close to that reported in the literature for elite athletes (71, 72). Tuo et al. (73) evaluated games from different countries at the FIFA World Cup and reported that players covered an average of 9.5 km per game and reached a maximum speed of 28 km/h. In our GPS analysis, the athletes covered 6 to 9.8 km and reached a maximum speed of 24.5 to 35.2 km/h, discarding the possibility that the results found occurred due to the low intensity of the games.

Our findings support that PP can be a substitute for WP. Although post-game CK and ALT values were lower in the PP group, the difference was only found in the delta analysis. Furthermore, CMJ and VAS were not significantly different between groups, indicating that the metabolic alterations found were not sufficient to modify the response to fatigue or muscle damage in a short period. Therefore, it is not possible to state that supplementation with PP was superior to WP in the recovery of athletes.

The relatively small sample size and no placebo control group indicate the limitations of the study. On the other hand, the type of study (a randomized, double-blind, crossover trial), period of supplementation, assessment of dietary intake, and similar levels of physical performance among athletes are some of the strong points of the study. Future studies that have larger sample sizes and verify the effect of these supplements with more time intervals or more consecutive games may contribute to elucidating whether differences exist between plant and animal proteins in modulating biochemical and metabolomic profiles associated with physical exercise.

## Conclusions

The results obtained suggest that both types of protein supplementation may have contributed to the reduction in cellular damage, with no metabolic alteration compatible with cell damage after 72 h of the game. Although the levels of LA, CK, sarcosine, and 3-hydroxybutyrate were lower in PP when compared with WP supplementation, the biochemical markers show a difference only in delta values and CMJ and VAS have no significant difference. Thus, these findings may not justify the preferred choice of a particular type of protein. However, PP can substitute for WP without compromising athletes' recovery or performance.

## Data availability statement

The original contributions presented in the study are included in the article/Supplementary material, further inquiries can be directed to the corresponding author.

## Ethics statement

The studies involving humans were approved by the University Hospital of the Federal University of Rio de Janeiro. The studies were conducted in accordance with the local legislation and institutional requirements. The participants provided their written informed consent to participate in this study.

## Author contributions

LL: conceptualization, methodology, validation, formal analysis, research, writing—original draft, writing—review and editing, and visualization. TF and VB: research, writing—original draft, writing—review and editing, and visualization. FC: formal analysis, investigation, writing—review and editing, and visualization. AV: statistical analysis, research, writing—original draft, writing—review and editing, visualization, and supervision. AP: conceptualization, methodology, statistical analysis, research, writing—original draft, writing—review and editing, supervision, project administration, and acquisition of funding. All authors contributed to the article and approved the submitted version.
