# Supplementary material for: Comparison of the effects of pea protein and whey protein on the metabolic profile of soccer athletes: a randomized, double-blind, crossover trial
Source: Front Nutr. 2023 Sep 22;10:1210215. doi: 10.3389/fnut.2023.1210215 (PMC10556705; doi:10.3389/fnut.2023.1210215)
Supplement: Supplementary file 1 [file Table_1.pdf]

Supplementary Material

Comparison of the effects of pea protein and whey protein on the metabolomic profile of soccer athletes: a randomized, double-blind, crossover trial

Luiz Lannes Loureiro, Tathiany Jéssica Ferreira, Fábio Luiz Candido Cahuê, Victor Zaban Bittencourt, Ana Paula Valente, Anna Paola Trindade Rocha Pierucci*

*** Correspondence:**
Anna Paola Trindade Rocha Pierucci
pierucci@nutricao.ufrj.br

Supplementary Table 1. Nutritional composition of Whey Protein (WP) or Pea Protein (PP) for 100g of powder.

	WP	PP
**100g of powder**		
Energy (kcal)	384.4	366.7
Carbohydrate (g)	6.8	0.7
Protein (g)	81.6	83.3
Lipid (g)	3.4	4.0
**On 100 g of protein**		
Aspartic Acid (g)	11.0	11.4
Glutamic Acid (g)	17.7	16.6
Alanine (g)	5.4	4.3
Arginine (g)	0.1	8.7
Cysteine (g)	2.5	1.0
Phenylalanine (g)	3.6	5.5
Glycine (g)	2.2	4.1
Histidine (g)	2.2	2.5
Isoleucine (g)	5.8	4.5
Leucine (g)	8.6	8.4
Lysine (g)	10.3	7.1
Methionine (g)	2.1	1.1
Proline (g)	6.6	4.5
Serine (g)	4.8	5.1
Tyrosine (g)	3.0	3.8
Threonine (g)	7.0	5.6
Tryptophan (g)	1.7	1.0
Valine (g)	5.2	5.0

PP: pea protein, WP: whey protein.
